# A Displaced Intrauterine Device Eroding Into the Sigmoid Colon

**DOI:** 10.7759/cureus.62238

**Published:** 2024-06-12

**Authors:** Savni Satoskar, Eva Zhang, David Fan, Yonas Teklu, Cesar Riera Gonzalez, Sneha George Teressa, Anshuman Sikka, Harsh R Parikh, William Lois

**Affiliations:** 1 General Surgery, BronxCare Health System, Bronx, USA; 2 Surgery, St. George's University, St. George's, GRD; 3 Surgery, BronxCare Health System, Bronx, USA; 4 School of Medicine, St. George's University, St. George's, GRD

**Keywords:** iud embedded in the mesocolon, laparoscopy for iud removal, iud complications, minimally invasive iud removal, displaced iud

## Abstract

A 36-year-old female presented to the gynecology office eight weeks after the placement of a ParaGard intrauterine device (IUD). Upon gynecologic examination, the strings of the IUD were not found. Magnetic resonance imaging was performed which reported the IUD embedded in the sigmoid colon. Initial diagnostic laparoscopy was done without bowel preparation and revealed an IUD embedded within the sigmoid colon and mesocolon. Colonoscopy did not reveal any breach of the colonic lumen. A second diagnostic laparoscopy was planned with the robotic-assisted technique after bowel preparation. Intraoperative findings during the second operation identified the IUD embedded in the antimesenteric side of the sigmoid colon with surrounding scar tissue to the uterus. The IUD was sharply freed using robotic scissors and the resulting serosal defect was sutured in layers with buttress made of appendices epiploica. The patient recovered well and had an excellent outcome. Our article highlights the minimally invasive method of dealing with a displaced IUD. The use of the robotic technique was helpful in our case to achieve an excellent outcome.

## Introduction

Intrauterine devices (IUDs) are a common choice for contraception due to their ease of use and overall safety. However, when an IUD is displaced, it may cause complications due to erosion into the bowel or the bladder. Uterine perforation from IUD insertion is a rare but recognized complication. In a large multisite cohort study published in 2022, it was found that the incidence of uterine perforation in the non-postpartum group was only 0.29%, while the incidence in the postpartum group ranged from 2.73% to 6.71% [1]. Common sites for IUD perforation include the cul-de-sac, omentum, and within the broad ligament [2]. A less common site for perforation that causes serious complication is the intestinal tract. Signs and symptoms of intestinal involvement can include fever, abdominal pain, obstruction, GI bleed, and increased WBC count [2]. Of the 41 intestinal cases reviewed by Zakin et al., urgent surgical intervention was needed in 19 patients [2]. We present a case where a displaced IUD embedded in the sigmoid colon was safely removed using the robotic approach with an excellent patient outcome.

## Case presentation

**Figure 1:**
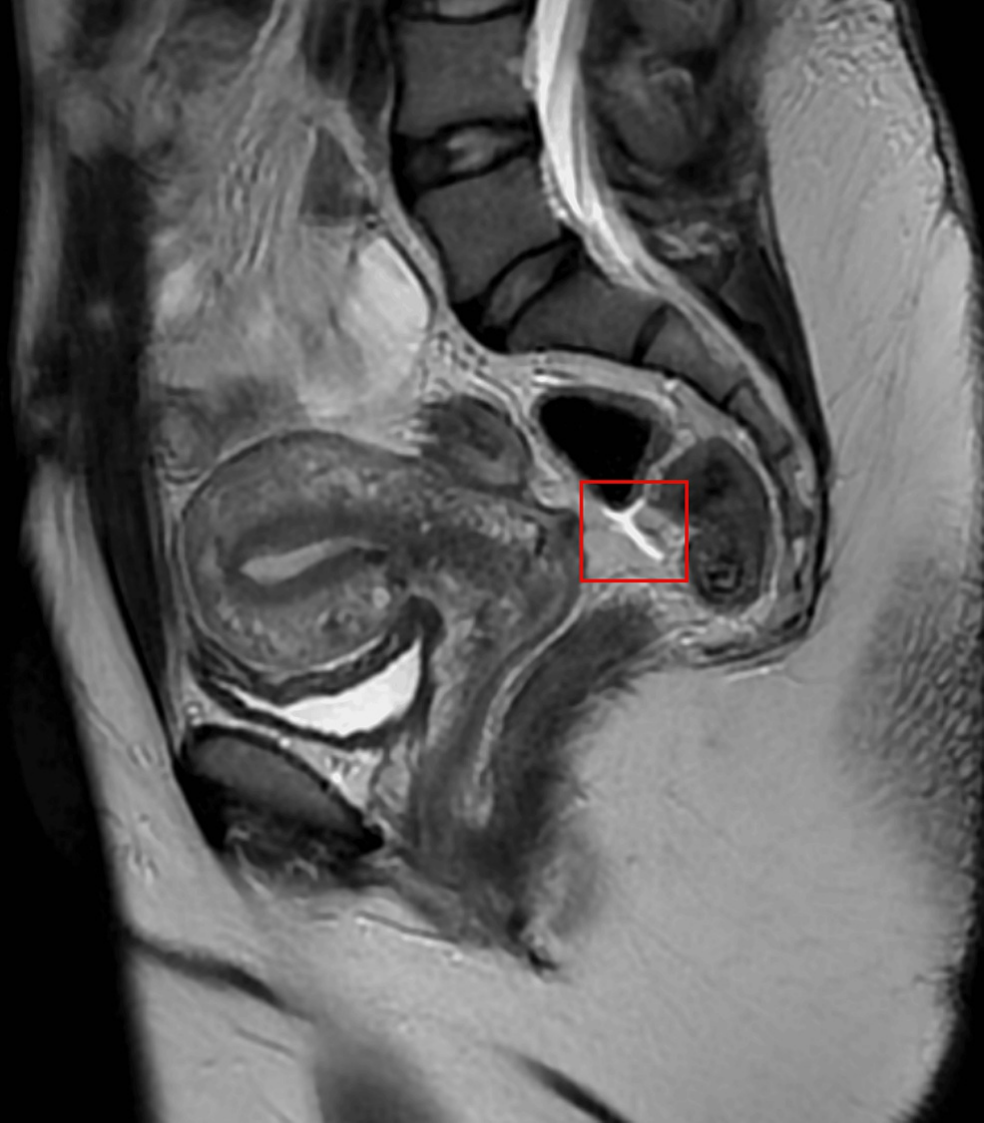
Abdominal MRI to Demonstrate the Lodged Intrauterine Device (IUD) Sagittal view of an abdominal magnetic resonance image (MRI) with contrast to highlight an intrauterine device (IUD) that is found to be embedded in the sigmoid colon, visualized in the red square.

A 36-year-old female presented to the gynecology office eight weeks after placement of a ParaGard intrauterine device. Upon gynecologic examination, the strings of the IUD were not found. A pelvic ultrasound was obtained which showed the IUD. She was advised to follow up in 6 to 12 months with a repeat ultrasound which reported that the IUD had migrated posterior to the cervix and was not within the uterine cavity. Magnetic resonance imaging (MRI) was performed which reported that the IUD was embedded in the sigmoid colon (Figure 1).

She remained asymptomatic and was using no alternative method of contraception. The decision was made by the Gynecology team to perform diagnostic laparoscopy with the goal of localizing and removing the IUD. Intraoperatively, the IUD was localized and was found to be embedded within the sigmoid colon. Intraoperative General Surgery consultation was requested. The patient had not been provided with a preoperative colon preparation and thus the recommendation was to postpone the removal of the IUD at that time. Postoperatively, she was referred to the surgery clinic for further evaluation. For operative planning, colonoscopy was requested which did not reveal that the IUD had migrated into the lumen of the sigmoid colon.

**Figure 2:**
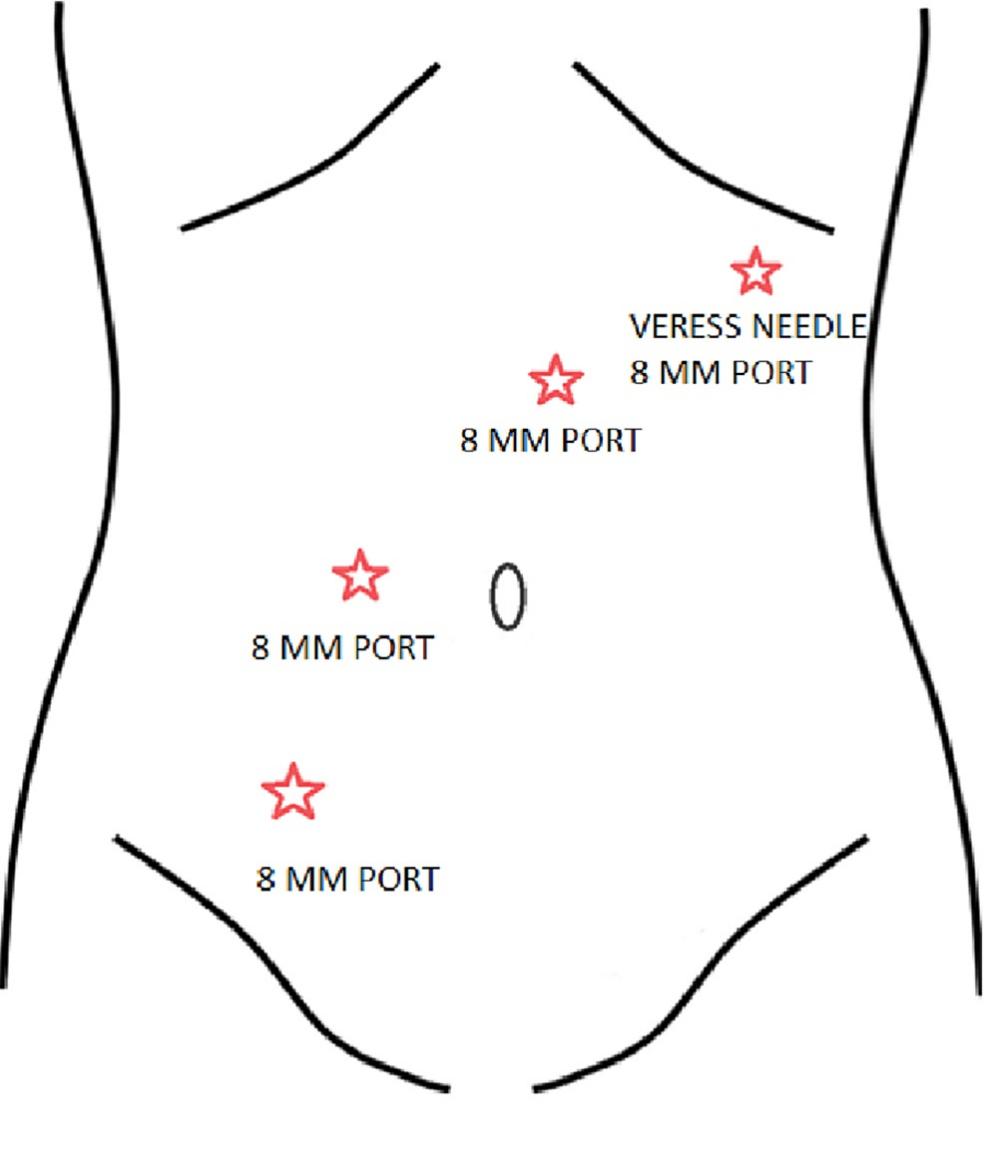
Port Placement Outline for Performed Diagnostic Laparoscopy The da Vinci Robot Port Placement outline running across the body, each red star indicating the approximate placement for the labeled port insertion.

The patient was prescribed appropriate preoperative colon preparation and planned for a second diagnostic laparoscopy by the General Surgery team. The operation was planned using the da Vinci Xi robot with port placement as shown in Figure 2.

Pneumoperitoneum was obtained through a left subcostal Veress needle placement, followed by a 5 mm optical port in the epigastrium to the left of the midline. Additional 8 mm robotic ports were placed in the right lower quadrant, through the Veress needle insertion site, and right mid abdomen. The initial 5 mm optical port was upsized to an 8 mm robotic port.

Intraoperative findings during the second operation identified the IUD embedded in the antimesenteric side of the sigmoid colon with surrounding scar tissue to the uterus.

**Figure 3:**
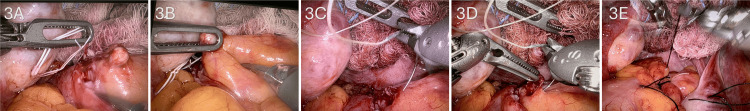
Intraoperative Localization of the IUD and Repair of the Sigmoid Colon A and B: The localization and identification of the dislodged intrauterine device (IUD) in the antimesenteric aspect of the sigmoid colon, visualized by the free-floating strings in the peritoneal cavity. C and D: Following dissection and removal of the IUD from the sigmoid colon, the serosal defect was repaired in layers of seromuscular sutures using polyglactin (Vicryl) and silk. E: The final repair consisted of a layer of interrupted polyglactin sutures with an overlying layer of silk sutures using the appendices epiploicae as a buttress.

The IUD was carefully dissected free from the sigmoid colon using sharp dissection with robotic scissors. The IUD was removed in one piece and out of the abdominal cavity through the right lower quadrant 8mm port. A serosal defect, approximately 4 cm long, was repaired in layers using interrupted size 3-0 polyglactin (Vicryl) seromuscular sutures, with overlying buttress of epiploic fat using size 3-0 silk interrupted sutures (Figure 3). The postoperative course was uneventful and the patient recovered well from the operation.

## Discussion

Uterine perforation from IUD insertion is a rare but recognized complication. According to the current guidelines of American College of Obstetricians and Gynecologists, when IUD strings are not visualized, endocervical cytobrush sweeps can be performed to achieve visualization [3]. If not successful, an ultrasound should be obtained after pregnancy is ruled out. If the IUD is not visualized in the uterus on ultrasound, X-ray/MRI should be obtained [3]. Surgical approaches such as laparoscopy and laparotomy can be considered after localization of the IUD within the body [3].

Esposito et al. proposed two possible mechanisms of uterine perforation, the first being traumatic perforation during IUD insertion, and the second due to gradual erosion through the myometrium [4]. In our patient, the IUD strings were not visualized at the first checkup following IUD insertion, favoring the first proposed mechanism by Esposito et al. To classify the degree of uterine perforation of our patient, we referred to the analysis of the literature by Zakin et al. Complete perforation is defined by penetration of the IUD through the endometrium, myometrium, and serosa [2]. Partial perforation is defined by penetration to the myometrium [2].

According to the literature review by Zakin et al., the most common location for complete uterine perforation is the Pouch of Douglas [2]. Anatomical variations such as flexion and version of the uterus play a role in the risk of uterine perforation. The more acute the angle of flexion is, the more likely perforation through the posterior uterine wall upon IUD insertion will occur [5]. If a mismatch exists between flexion and version of the uterus, the risk of uterine perforation will also increase [5]. Although the majority of IUD insertions now are done without additional imaging, we recommend ultrasound guidance, should clinicians encounter unexpected uterus orientation.

Multiple studies were done to investigate the rate of uterine perforation between copper IUDs and levonorgestrel-containing IUDs and no studies showed a significant difference between the two [5-7]. Despite similarity in perforation rates, copper IUDs are associated with a significantly higher incidence of dense adhesions [5]. Upon the first diagnostic laparoscopy done by the Gynecology team, our patient was noted to have dense adhesions between the sigmoid colon and the cul-de-sac, which were subsequently lysed. As pointed out by Kho et al., there was no correlation between pain symptoms and the presence or absence of adhesions [5]. Our patient was asymptomatic since the day of IUD insertion despite the dense adhesions seen on laparoscopy.

We believe the use of the da Vinci Xi robot was extremely helpful in this case. In the systemic review to compare laparoscopic and open surgery in intraperitoneal IUD removal by Mosley et al., 93% of the cases were planned to be laparoscopic [8]. 22.5% of the laparoscopic cases were converted to laparotomies [8]. The rate of conversion to open operation is related to the location of the migrated IUD and the degree of embedment of the IUD [8]. The most challenging maneuvers in laparoscopic surgery are intracorporeal knot tying and suturing [9]. The long, rigid instruments of laparoscopic surgery usually do not have intricate wrist joint at the end effector, whereas the robots have articulating mechanical wrists that allow enhanced dexterity and degree of freedom [10]. In the case report of laparoscopic removal of the IUD in the small intestine by Rahnemai-Azar et al., a small section of the bowel was resected and anastomosed [11]. Utilization of a wound protector/retraction device was helpful in performing an extracorporeal anastomosis [11]. In our patient, IUD embedment only involved the serosa of the sigmoid colon, but repair of the defect still required repair in layers in a Lembert fashion. The robot was also helpful in negating the fulcrum effect created by the abdominal wall on the end effector as typically seen in manual laparoscopy.

Most cases of uterine perforations are asymptomatic, but physicians should be aware of the serious complications that can result. Utilization of ultrasound upon unexpected uterus orientation and timely follow-ups after IUD insertion are recommended. Surgical removal of the migrated IUD in this patient was warranted due to the possibility of sigmoid colon perforation and related complications.

## Conclusions

Migrated IUDs are a serious problem, and due to the widespread use of IUDs, this is an occurrence that is often seen. In cases of IUDs that are embedded in the colon, operative intervention is warranted to avoid the risk of erosion into the colon. The use of the robotic approach facilitated successful removal of the displaced IUD in our case without any complications.
